# Demographics, Characteristics, and Outcomes of Male Breast Cancer Patients at the Methodist Health System, Dallas, USA

**DOI:** 10.7759/cureus.49394

**Published:** 2023-11-25

**Authors:** Huy Q Nong, Darcy Eastwood, Kimberly Rodriguez, Vichin Puri

**Affiliations:** 1 Internal Medicine, Methodist Dallas Medical Center, Dallas, USA; 2 Geriatrics, Baylor College of Medicine, Houston, USA; 3 Surgery, Methodist Dallas Medical Center, Dallas, USA; 4 Oncology, Methodist Dallas Medical Center, Dallas, USA

**Keywords:** medical oncology, oncology, surgical oncology, breast oncology, male breast cancer, breast cancer research

## Abstract

Breast cancer (BC) is the most common cancer in women and has been extensively studied; however, male BC (MBC) is rare with limited clinical data. Treatment options for MBC are extrapolated from clinical studies in BC in women and have traditionally excluded MBC cases. Over the past decade, an increase in the incidence of MBC has been seen. The purpose of this study is to comprehensively analyze the clinical, pathological, and treatment-related characteristics of MBC cases within our institution's database. MBC cases from 2010 to 2021 at Methodist Dallas Medical Center (MDMC), Dallas, USA, were reviewed retrospectively from the electronic health record and database, and clinical information was obtained. During this time period from 2010 to 2021, there was a total of 1,784 cases of BC with only eight cases (0.45%) consisting of MBC. In our cohort, 75% of MBC cases had a family history of cancer in a first-degree relative. Additionally, 100% of all MBC cases are hormone receptor-positive. No cases of MBC had HER2/neu over-expression. Fifty percent of our MBC patients were diagnosed with locally advanced tumors or metastatic disease. The overall survival (OS) of MBC in our study was 72%.

## Introduction

Breast cancer (BC) is the leading cause of cancer and cancer-related death among women worldwide and has been extensively studied. However, male BC (MBC) is a rare entity, accounting for less than 1% of all BC cases [1-4]. Treatment options for MBC are extrapolated from clinical trials among women with BC and men have traditionally been excluded from such studies [1,2]. To date, there are no large clinical trials exclusively for MBC [1].

The lifetime risk of BC for a man is 1:1,000, as compared to 1:8 for women [2]. There was an increase in worldwide cases of MBC from 8,500 in 1990 to 23,100 cases in 2017 [2]. Such a rise in the incidence of MBC may be explained by the increase in longevity of the general population, as well as a growing prevalence of obesity, both of which are known risk factors for BC in men [2]. The recent increasing incidence of MBC has led to further interest by the scientific community in better understanding this malignancy. 

Other risk factors for BC among men and women include family history and germline mutations. A family history of BC or ovarian cancer confers a higher risk for developing BC in men than it is in women [4]. A family history of BC increases the risk of MBC (relative risk (RR) 2.5), and 20% of men with BC have a first-degree relative of BC. Mutations in BRCA genes are among the most clearly established risk factors for BC in men [1]. BRCA1 and BRCA2 are tumor-suppressor genes involved in DNA repair. Germline BRCA2 mutations have been previously reported in 4-14% of patients with MBC, whereas BRCA1 mutations are less frequent, occurring up to 4% [4]. BRCA1 and BRCA2 germline mutations confer an estimated lifetime risk of developing BC in men of 1-6% and ~7%, respectively [4]. Other notable germline mutations that confer an increased risk of developing BC in men include CHEK2, PALB2, and CYP171 [4].

BC both in men and women is an age-related disease, with an incidence rate rising steadily with age [1]. Interestingly, MBCs are usually diagnosed at a later age of approximately five years older for men than for women [1,4]. Other important established risk factors for BC in men include radiation exposure, increased serum estradiol, obesity, and gynecomastia [1,4]. Changes in the hormone balances of estrogen to androgen, such as in Klinefelter’s syndrome, testicular abnormalities that result in testosterone deficiency, liver disease, obesity, and exogenous estrogen exposure are risk factors in the development of MBC [1,2,4].

Several studies have shown that the overall survival (OS) for MBC is significantly lower than for female BC [1,4,5]. Further understanding of the difference may deepen the contrast between male and female BCs, and, as a result, MBC may warrant different management and treatment strategies than women [6]. The aim of this study is to review the diagnosis and management of MBC at our institution and provide important relevant clinical information that may inform the future of MBC care.

## Materials and methods

Design and oversight

We conducted a case series review in which all MBC cases treated at our institution were included. The study was conducted in accordance with regulatory requirements and the International Conference on Harmonization Good Clinical Practice guidelines. The protocol, 015.MTP.2023.A, was approved by the Institutional Review Board (IRB) and Ethics Committees at the Methodist Dallas Medical Center (MDMC), Dallas, USA. Due to the retrospective nature of the study, a full waiver of the informed consent process was obtained by the Methodist Health System IRB. The authors wrote the manuscript with the assistance of a medical writer. All authors had full access to relevant data, vouch for the completeness and accuracy of the data and for adherence to the trial protocol, and had final responsibility for the content of the manuscript and for the decision to submit the manuscript for publication. This case series involves a retrospective review of all MBC cases diagnosed and treated at Methodist Dallas Medical Center (MDMC) over the time period of 2010-2021. The purpose of this study is to comprehensively analyze the clinical, pathological, and treatment-related characteristics of MBC cases within the hospital's database.

Patients

MBC cases from 2010 to 2021 were reviewed retrospectively from the electronic health record and database from Methodist Dallas Medical Center. Clinical information was stored on a secure server with patients' names and information de-identified. Inclusion criteria were male patients with early-stage BC, locally advanced, and metastatic BC. Ductal carcinoma in situ (DCIS) was included. Diagnosis was made based on biopsy-proven histopathology.

Variables and endpoints

The primary objective was to review clinical information regarding MBC treated at our institution. A complete list of eligibility criteria is provided in the protocol. The following data elements were collected:

Patient demographic information: Age at diagnosis, race, and family history of BC

Clinical characteristics: Date of diagnosis, presenting symptoms, physical examination findings, and genetic testing

Pathological information: Histological subtype, tumor size, histological grade, hormone receptor status (estrogen and progesterone), HER2/neu status, and lymph node involvement

Staging and prognostic factors: AJCC tumor stage, tumor-node-metastasis (TNM) classification, and any relevant prognostic factors, such as Ki-67 index

Treatment modalities: Type of surgery (e.g., mastectomy or lumpectomy), axillary lymph node dissection or sentinel lymph node biopsy, radiation therapy, chemotherapy, hormonal therapy, and targeted therapy

Follow-up data: Information on disease recurrence, overall survival, and adjuvant treatments

Statistical analysis

Descriptive statistics was used to summarize patient demographics, clinical characteristics, and treatment modalities. Demographic and clinical characteristics, as well as treatment modalities, were summarized. OS was defined as the time from the date of diagnosis to the date of death.

## Results

Patient characteristics

Demographic and disease characteristics at baseline were recorded (Table 1). Between 2010 and 2021, a total of seven male patients were diagnosed with infiltrating ductal carcinoma (IDC) of the breast (Table 2). One patient was diagnosed with DCIS (Table 2). The median age at diagnosis was 71 years old. Half of the patients were Caucasian, while the other half were Black (Table 1). Of the total population, two had distant metastasis to the bone, while the rest were disease-free after treatment (Table 2).

**Table 1:** Demographics, oncology history, and family history

No	Age at Diagnosis (y.o.)	Race	Oncologic Medical History	Family History
1	68	Caucasian	Neuroendocrine tumor of duodenum	Breast cancer in mother and sister; prostate cancer in father
2	54	Black	Unremarkable	Breast cancer in mom and sister
3	63	Caucasian	Unremarkable	Unremarkable
4	75	Caucasian	Unremarkable	Breast cancer in mother; pancreatic cancer in father, colon cancer in brother
5	78	Black	Unremarkable	Breast cancer in daughter and sister; prostate cancer in both brothers
6	74	Black	Unremarkable	Unknown malignancy in the father
7	77	Caucasian	Unremarkable	Melanoma in mother
8	67	Black	Pancreatic adenocarcinoma	Unremarkable

**Table 2:** Patient's clinical staging, tumor receptor status, and treatment summary *Patient had concurrent pancreas adenocarcinoma. MRM = Modified radical mastectomy; TM = Total mastectomy; SLND = Sentinel lymph node dissection; ALND = axillary lymph node dissection

No	Diagnosis	Staging	Hormone Receptor	HER2/neu	Genetic Testing	Treatment Summary	Recurrence	Clinical Status
1	Invasive Ductal Carcinoma	Stage IIA cT2 cN0 cM0	ER+/PR+	HER2­-	BRCA negative	MRM without removal of contralateral breast with SLN + ALND, radiation therapy, systemic chemotherapy and tamoxifen, leuprorelin, letrozole, Palbociclib	Distant metastasis to Left 9^th^ rib	Deceased
2	Invasive Ductal Carcinoma	Stage IV cT4 cN1 cM1	ER+/PR+	HER2­-	BRCA negative, VUS in APC gene (c.1825G>A), VUS in POLE Gene (c.2089C>T)	Neoadjuvant paclitaxel, MRM without removal of contralateral breast + ALND, Radiation therapy, tamoxifen had RT then started Capecitabine, Zoledronic acid Tamoxifen, switched to letrozole and Palbociclib	Disease Free, no recurrence	Alive
3	Invasive Ductal Carcinoma	Stage IA cT1 cN0 cM0	ER+/PR+	HER2­-	None	No systemic therapy, surgery, or radiation. Only tamoxifen	Disease free, no recurrence	Alive
4	Invasive Ductal Carcinoma	Stage IIIB cT4b cN1 cM0	ER+/PR+	HER2­-	BRCA negative	Total mastectomy with SLND + ALND followed with radiation and adjuvant chemotherapy, tamoxifen	Distant metastasis to spine	Deceased
5	Invasive Ductal Carcinoma	Stage IA cT1c cN0 cM0	ER+/PR+	HER2­-	BRCA negative	TM with SLN biopsy followed by radiation therapy, adjuvant chemotherapy and tamoxifen	No recurrence	Alive
6	Invasive Ductal Carcinoma	Stage IIIB cT4b cN0 cM0	ER+/PR+	HER2­-	None	Neoadjuvant chemotherapy, TM with SLN biopsy, adjuvant chemotherapy and radiation, tamoxifen	No recurrence	Alive
7	Ductal Carcinoma In situ*	Stage 0 cTis cN0 cM0	ER+/PR+		none	TM with SLN biopsy, tamoxifen		Alive
8	Invasive Ductal Carcinoma	Stage IIIB cT4c cN1 cM0	ER+/PR+	HER2­-	None	Neoadjuvant chemotherapy*		Alive

Diagnosis, staging, and tumor characteristics

MBC cases are presented at various stages at diagnosis (Table 2). One case was DCIS (Stage 0). Three cases were diagnosed as early-stage disease (Stages IA-IIA) disease (Table 2). Four cases were diagnosed as locally advanced disease (Stages IIB-IIIB) disease (Table 2). Only one case was diagnosed as metastatic (Stage IV) disease (Table 2). All MBC cases were estrogen receptor-positive, progesterone receptor-positive, and HER2 receptor-negative (Table 2).

Genetic testing

Within the MBC population, only half of the patients underwent genetic testing for germline mutations (Table 2). No MBC had detectable BRCA mutations or other notable germline mutations including CHEK2, PALB2, and CYP17 (Table 2). Genetic testing in one patient revealed two variants of unknown significance (VUS) in the APC gene (c.1825G>A) and the POLE gene (c.2089C>T) (Table 2).

Family history

In our cohort, six patients (75%) with MBC had a family history of cancer in a first-degree relative (Table 1). Four patients had a first-degree relative (FDR) with BC (Table 1). In one case, the patient had a family history of prostate cancer in his father and BC in both his sister and mother (Table 1). In a second case, the patient had a mother and a sister both with BC (Table 1). A third MBC patient had BC in only his mother (Table 1). A fourth case had both a daughter and sister with BC (Table 1).

Treatment history

MBC treatments were based on presenting clinical stages and tumor characteristics. Treatment paradigms were mostly consistent across all MBC cases with surgical mastectomy, followed by adjuvant radiation therapy, with or without adjuvant chemotherapy, followed by endocrine therapy. Six of the eight cases (75%) underwent mastectomy with a sentinel lymph node (SLN) biopsy (Table 2). The combination of the aromatase inhibitor with a CDK4/6 inhibitor was used for one patient who had metastatic disease with the addition of bisphosphonate for bone metastasis (Table 2).

Outcomes

Two cases of MBC had a recurrence of the disease with metastasis to the bone after initial treatment (Table 2). One patient had osseous metastasis to the left ninth rib (Table 2). Another patient had osseous metastasis to the spine. The other six cases were disease-free at the time of analysis (Table 2). Both patients who had distant metastatic bone lesions died due to their cancer. The OS of our MBC cohort was 72% (Table 2).

## Discussion

Among all BC patients treated at our institution from 2010 to 2021, only eight cases (0.45%) were MBC, including one case that was DCIS. The majority (50%) of our MBC cases were diagnosed at a locally advanced stage or later suggesting that MBC at our institution was diagnosed at a predominately late stage. This is consistent with previous studies reporting that MBCs are diagnosed at more advanced stages compared to female BC [1].

All of our MBC cases (100%) were hormone receptor-positive. MBCs have been found to be more hormone receptor-positive compared to female breast cancers in other studies (estrogen receptor (ER) positivity 91-95% vs. 76-78% in men and women, respectively) [1,4]. None of the MBC cases in our cohort were found to have HER2 over-expression, which was also consistent with previous studies showing that MBCs are less likely to express HER2 receptors compared to FBC (9% vs. 15%, respectively) [5,7]. HER2-positive breast tumors are characterized as more aggressive, having high rates of cell proliferation, and higher risks of recurrence. However, HER2-receptor positivity offers opportunities for additional treatment options with anti-HER2-directed therapy, such as trastuzumab and pertuzumab, which selectively binds to HER2 receptors and blocks the signaling and proliferation of tumor cells. HER2-directed therapy confers significant improvement in survival outcomes. However, MBCs are less likely to be HER2-positive, and therefore HER2-directed therapy would not be indicated, further limiting treatment options in this group of patients.

The MBC cases in our cohort displayed a strong genetic component. Fifty percent of our MBC cases had a family history of BC in a first-degree female relative, and 75% of our MBC cases had a family history of malignancy of any kind, suggesting that genetic factors play a large role in the development of BC in men, which is consistent with other studies [1-2]. A family history of BC in a first-degree relative is an important risk factor for MBC, noted as early as 1892 [2]. Having a first-degree relative with BC confers a two- to threefold increase in the risk of having MBC [2] (RR = 1.92, 95% CI: 1.19-3.09) [8-10]. Population studies have shown that approximately 20% of all MBC patients have a history of BC in a first-degree relative [2,8], which is higher than that observed in FBC (~7%) [2,11-14]. Half of the MBC patients in our study did not undergo genetic testing. The reasoning was likely multifactorial but unclear at the time of analysis. Current guidelines recommend that all male patients with BC should be offered genetic counseling and genetic testing for germline mutations [9,12,13,15]. In addition to BRCA1 and BCRA2 genes, other genes, such as CHEK2, PALB2, and PTEN, are less common but also confer an elevated risk of breast cancer among men [11,13-17]. Over 20% of MBC patients carry an identifiable inherited risk factor for breast cancer [17]. Identification of an inherited risk factor may influence screening recommendations for other related cancers and lead to the testing of family members for inheritable genes [17].

Current guidelines do not recommend screening mammography for male patients, even for those who are at increased risk of developing BC. However, given the significant increase in the risk of developing MBC in male patients with a family history of BC or in male patients with a notable germline mutation, these healthy male patients may benefit from routine screening with imaging of mammography or ultrasound in addition to the current standard of clinical breast examination [9,11]. An effective and targeted screening protocol for at-risk men may improve overall outcomes in MBC.

Treatment of MBC at our institution was largely based on guidelines for BC in women. Early-stage MBC underwent an upfront mastectomy, followed by adjuvant radiation and hormonal therapy with tamoxifen. Locally advanced MBCs at our institution were treated with neoadjuvant chemotherapy, followed by mastectomy and adjuvant radiation with tamoxifen. Despite our best efforts, recurrence occurred in 25% of our MBC patients. MBC is a rare disease with no randomized control trials to support specific diagnostic or treatment options.

The strengths of this study are that it reports on many unique features of MBC including epidemiological factors, genetics, tumor histology, treatments, and clinical outcomes. Taken as a whole, the study provides a strong overview of how MBC was evaluated and treated over a span of a decade at our institution. Within these data points, we highlight some contrasting features between MBC and FBC, which are clinically relevant. The rich clinical data allow for the observation of trends in MBC patterns, as well as the potential for improved treatment and outcomes in the future as this rare entity becomes better understood.

While this case series on male breast cancer at our institution offers valuable insights into the clinical characteristics and treatment outcomes of this relatively rare condition, it is important to acknowledge several limitations that may impact the generalizability and interpretation of our findings. Firstly, the retrospective nature of the study introduces inherent biases, as it relies on existing medical records and data, potentially leading to incomplete or missing information. Additionally, the single-institution focus of the study may limit the diversity of cases and treatments, potentially affecting the generalizability of the results to a broader population. Furthermore, the relatively small sample size inherent to MBC cases may restrict the statistical power for certain analyses, making it challenging to detect significant associations or differences. Lastly, the study's findings are contingent upon the quality and accuracy of medical record documentation. Despite these limitations, this case series serves as a valuable foundation for understanding MBC within our institution and provides a platform for future research in this under-explored area of oncology. The National Cancer Database (NCDB) reports that the incidence of MBC was 7.2% in 2004 and has risen up to 10.3% in 2014 [6]. The rationale for this finding is multifactorial but includes a rising rate of obesity and older age of the general population [2]. As MBC cases continue to increase worldwide, more research is necessary to properly evaluate and manage MBC as well as to improve outcomes. In our study, regardless of the small sample size, we report important clinical information on MBC.

## Conclusions

In conclusion, the rarity of MBC makes it challenging to study this population in either randomized clinical trials or cohort studies. This study highlights significant clinical information on MBC treated at one institution. Importantly, our study showed that MBCs in our center had a strong genetic component with 75% of cases having a family history of cancer, although no specific germline mutations were found in our study. The OS was low at 72%. All MBCs should undergo genetic testing according to current guidelines, which was not done at our institution for various reasons. MBC incidence has been increasing at our institution, which is consistent with the trend in the general population. Our future aims include expanding our cohort to include MBC data from our partner institutions and having matched FBC controls to further study the differences between MBC and female BC.
